# Management of traumatic macrostriae in an undisplaced LASIK Flap

**DOI:** 10.22336/rjo.2024.61

**Published:** 2024

**Authors:** Mamta Singh, Nagendra Prasad, Bibhuti Prasanna Sinha

**Affiliations:** 1 Department of Ophthalmology, All India Institute of Medical Sciences, Rajkot, India; 2 Buddha Eye Care and Laser Centre, Patna, India; 3 Indira Gandhi Institute of Medical Sciences, Patna, India

**Keywords:** LASIK flap, macrostriae, macrofolds, flap stretching, LASIK = Laser in situ keratomileusis, OD = Right eye, OS = Left eye, BCL = Bandage contact lens

## Abstract

**Purpose:**

To present a case of traumatic late macrostriae of Laser in situ keratomileusis (LASIK) flap managed by flap lifting, stretching, and polishing.

**Material and method:**

A patient presented with a history of defective vision in his right eye following trauma with a rubber ball 10 days ago. He had undergone an uneventful LASIK surgery 4 years ago. Ocular examination showed visual acuity of 20/200, multiple parallel radiating folds in an undisplaced LASIK flap in the inferonasal quadrant, and sphincter tear. This case required an urgent surgical intervention. Epithelial debridement, flap lifting, gentle stretching, and irrigation were performed to smooth out the striae. A bandage contact lens was applied to ensure proper wound apposition.

**Results:**

The postoperative period was without complications, and the patient achieved a final visual acuity of 20/20.

**Discussions:**

The insufficient wound healing of the LASIK flap results in a cornea with compromised biomechanical strength. They remain susceptible to trauma for a long duration after surgery. Traumatic injury to these eyes can lead to late macrostriae formation, which results in visual deterioration. Cases of macrostriae presenting late require surgical debridement of epithelium, which keeps these folds fixed. It should be followed by flap irrigation and stretching to smooth these striae.

**Conclusions:**

Since LASIK wound healing is always incomplete, it is crucial to inform patients about the potential risk of trauma. Any traumatic flap injury requires thorough examination and proper management of these cases results in excellent visual gain.

## Introduction

Laser in situ keratomileusis (LASIK) has revolutionized the approach to refractive error correction. However, this procedure does not lack its distinct challenges, flap-related issues being particularly crucial. Flap striae are creases or puckers in the cornea following LASIK surgery [1]. They result from inadequate contour alignment, an uneven match between the flap edge and the peripheral epithelial ring, or flap displacement during the postoperative phase [2,3]. They can be in the form of microstriae, which are superficial asymptomatic folds involving the basement membrane of the corneal epithelium and/or Bowman’s layer, or macrostriae, which involve full thickness of flap and often lead to suboptimal vision. Most of these cases are seen in the early postoperative period [4]. We are reporting a case of post-traumatic late macrostriae of LASIK flap and its management.

## Case report

**Fig. 1:**
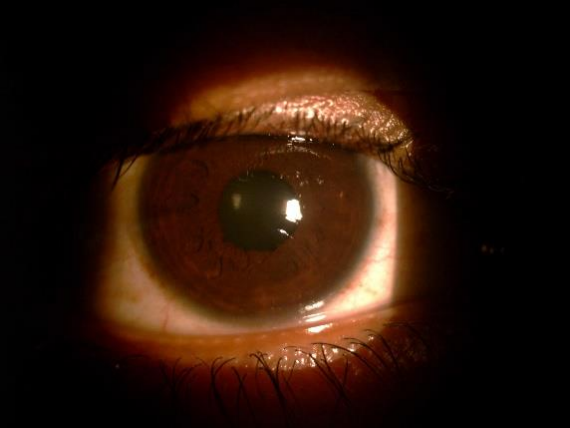
Macrostriae of LASIK flap

A young male in his 20s presented with a history of defective vision in his right eye (OD) following a soft rubber ball injury two weeks ago. The patient had undergone LASIK surgery without any complications four years ago and had been asymptomatic until the recent trauma. He was using topical moxifloxacin (0.5%) and loteprednol etabonate (0.5%) combination 4 times/day for the last 10 days prescribed after ocular trauma. The clinical records showed a pre-LASIK visual acuity of 20/20 in both eyes with -4.0D spherical in OD and -4.00 D spherical with -0.5D cylinder at 180 degrees in his left eye (OS). On examination, his best corrected visual acuity was 20/200 and 20/20 respectively in OD and OS. Both eye intraocular pressure was 14 mmHg by non-contact tonometer. Slit lamp examination of OD revealed multiple folds in the LASIK flap radiating from the inferonasal cornea towards the center of the pupil (**Fig. 1**). Anterior chamber had no cells or flare. The pupillary margin had sphincter tears at 5:30, 6:30, and 8 o’clock. The pupil was round and reactive to light reflex. Posterior segment evaluation was normal. The ocular examination of OS was normal, with a clear LASIK interface.

The patient was informed about the treatment options and visual prognosis and after getting the informed written consent, epithelial debridement and mechanical flap stretching were planned.

**Fig. 2:**
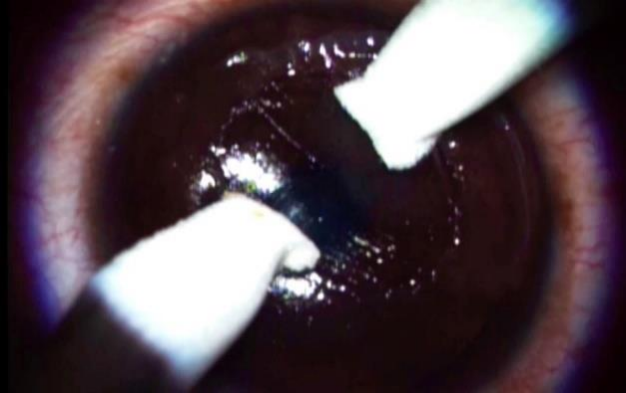
Flap stretching with two sponges

Under topical anesthesia, the epithelium was removed from the area over macrofolds and surrounding 2mm with the help of a Sinskey hook and dry Merocel sponge. The flap margin was defined with the help of a Sinskey hook, and it was separated from the stromal bed with a spatula. The flap was lifted without disturbing the nasal hinge and was hydrated with a balanced salt solution. With two Merocele sponges, it was simultaneously mechanically stretched in a direction perpendicular to the macrostriae and stabilized (**Fig. 2**). Once the folds were smoothed out, loose epithelial tags were removed, the flap and the stromal interface were irrigated, and proper flap positioning was ensured by gentle polishing. A bandage contact lens (BCL) was applied at the end of the surgery. The postoperative treatment included topical eye drops of 0.5% moxifloxacin hydrochloride, 1% prednisolone acetate (weekly tapering dose), and 0.5% carboxymethylcellulose - all 4 times daily. BCL was removed after epithelial healing on day 2.

## Results

**Fig. 3:**
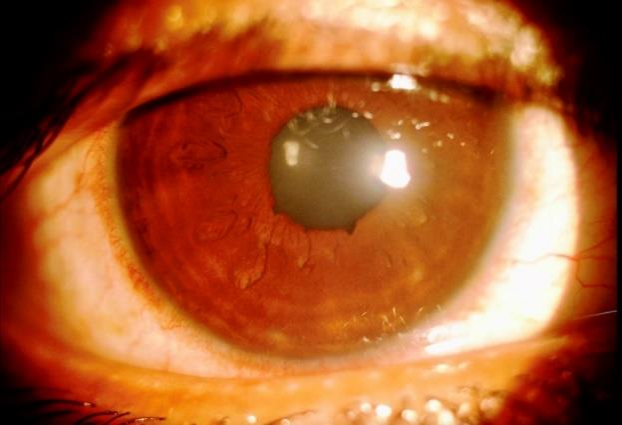
Postoperative picture at 1 week with no evidence of microstriae

1-week follow-up examination showed OD visual acuity of 20/20, with no evidence of macrostriae on the cornea (**Fig. 3**). The flap was in position and the interface was clear. The patient maintained a visual acuity of 20/20 until the last follow-up, of 1 year, from this surgery.

## Discussion

Lasik flap striae are established ocular complications involving 0.03% to 3.5% of operated eyes, however, only 0.2% to 1.5% of these cases necessitate interventions [1,5]. The early stability of LASIK flaps is maintained by capillarity, fiber interlacing, intracorneal suction, endothelial pumping, and ionic bonding [6]. Higher refractive error correction, intraoperative excessive flap manipulation, higher operating room temperature, flap drying, flap contracture, and incorrect wound apposition are risk factors for early flap misalignments (1–2%) [5,7,8]. Cases presenting beyond the first week or displaying late flap striae are primarily associated with trauma and flap displacement. This has been reported as late as 14 years of uneventful LASIK surgery by Holt et al. and Ting et al. and is linked to the limited and incomplete healing process of LASIK wounds [7,9]. The biomechanical strength of corneal tissue remains severely impaired after LASIK surgery, particularly in central and paracentral areas (approximately 2.4% as strong as normal corneal stroma) [10]. While it aids in preserving a clear optical axis and allows for lifting the flap for potential secondary procedures, it also makes the LASIK flap vulnerable to injury. Most of the late traumatic macrostriae have been reported with associated flap dislocation, similar to the above two reports [11-13]. In our case, the patient presented with macrostriae without flap displacement after 4 years of LASIK surgery following rubber ball trauma. In contrast to other cases, it was not associated with epithelial ingrowth as the flap integrity was maintained at the margin. Incomplete wound apposition is an important trigger for epithelial ingrowth. Ursea R and Feng MT reported a similar case of late traumatic flap striae, without concurrent flap dislocation [14].

The management of these cases with late macrostriae depends on the time of presentation after trauma and associated complications. The management option includes hypotonic saline irrigation, refloating, stretching and smoothing, flap massage, hyperthermia, the sandwich compression method, epithelium removal, Bowman’s layer phototherapeutic keratectomy, and flap suturing for recalcitrant cases [3]. Patients who present immediately after trauma can be treated by flop irrigating or gently lifting, repositioning it to its optimal location, or employing techniques such as flap-sliding or the double twist method [1,3]. The epithelium grows in the cervices of late-presenting cases like ours, forming fixed or established folds. These cases require epithelial removal to free these folds before ironing them back to their original position. Mechanical epithelial debridement can be achieved using surgical blades, a Merocel sponge, or any microsurgical blunt spatula. In our case, we preferred using a Merocel sponge because it allows for a non-traumatic, uniplanar removal of the epithelium, without causing damage to the underlying tissue. A gentle stroking pressure with a force vector directed away from the hinge further smooths these folds. Thorough irrigation of these flaps helps decrease the folds by creating temporary flap oedema and removing interface debris. In contrast to previously documented cases with flap dislocation and epithelial ingrowth, this case did not require the use of epithelial scraping from the stromal bed or under-surface of the flap, 50% ethanol or 70% isopropyl alcohol, or strict wound apposition by sutures, or application of tissue adhesive [12,15]. The application of BCL ensured the proper wound apposition. The final visual outcome of these cases is good unless complicated by postoperative infection, recurrent epithelial erosion, epithelial ingrowth, and diffuse lamellar keratitis [3]. In our case, the patient achieved 20/20 uncorrected visual acuity and maintained it until the last 1-year follow-up.

## Conclusion

LASIK wounds remain vulnerable to trauma for many years after surgery. Emphasizing the risk of trauma and the potential for reduced vision is essential for patient awareness. Late-onset LASIK macrostriae, especially those without flap dislocation, often go undiagnosed if not carefully examined. These cases, presenting late with macrostriae, need urgent surgical intervention and proper management, and good visual outcomes can be achieved.
